# Identification of genetic variants associated with diabetic kidney disease in multiple Korean cohorts via a genome-wide association study mega-analysis

**DOI:** 10.1186/s12916-022-02723-4

**Published:** 2023-01-11

**Authors:** Heejin Jin, Ye An Kim, Young Lee, Seung-hyun Kwon, Ah Ra Do, Sujin Seo, Sungho Won, Je Hyun Seo

**Affiliations:** 1 Institute of Health and Environment, Seoul National University, Seoul, Korea; 2 Division of Endocrinology, Department of Internal Medicine, Veterans Health Service Medical Center, Seoul, Korea; 3 Veterans Medical Research Institute, Veterans Health Service Medical Center, Jinhwangdo-ro 61-gil 53, Gangdong-gu, Seoul, Korea; 4 Interdisciplinary Program of Bioinformatics, College of National Sciences, Seoul National University, Seoul, South Korea; 5 Department of Public Health Science, Graduate School of Public Health, Seoul National University, Seoul, Korea; 6 RexSoft Corps, Seoul, Korea

**Keywords:** Diabetic kidney disease, GWAS, Genetic variants, Prediction, Microvascular complications

## Abstract

**Background:**

The pathogenesis of diabetic kidney disease (DKD) is complex, involving metabolic and hemodynamic factors. Although DKD has been established as a heritable disorder and several genetic studies have been conducted, the identification of unique genetic variants for DKD is limited by its multiplex classification based on the phenotypes of diabetes mellitus (DM) and chronic kidney disease (CKD). Thus, we aimed to identify the genetic variants related to DKD that differentiate it from type 2 DM and CKD.

**Methods:**

We conducted a large-scale genome-wide association study mega-analysis, combining Korean multi-cohorts using multinomial logistic regression. A total of 33,879 patients were classified into four groups—normal, DM without CKD, CKD without DM, and DKD—and were further analyzed to identify novel single-nucleotide polymorphisms (SNPs) associated with DKD. Additionally, fine-mapping analysis was conducted to investigate whether the variants of interest contribute to a trait. Conditional analyses adjusting for the effect of type 1 DM (T1D)-associated HLA variants were also performed to remove confounding factors of genetic association with T1D. Moreover, analysis of expression quantitative trait loci (eQTL) was performed using the Genotype-Tissue Expression project. Differentially expressed genes (DEGs) were analyzed using the Gene Expression Omnibus database (*GSE30529*). The significant eQTL DEGs were used to explore the predicted interaction networks using search tools for the retrieval of interacting genes and proteins.

**Results:**

We identified three novel SNPs [rs3128852 (*P* = 8.21×10^−25^), rs117744700 (*P* = 8.28×10^−10^), and rs28366355 (*P* = 2.04×10^−8^)] associated with DKD. Moreover, the fine-mapping study validated the causal relationship between rs3128852 and DKD. rs3128852 is an eQTL for *TRIM27* in whole blood tissues and *HLA-A* in adipose-subcutaneous tissues. rs28366355 is an eQTL for HLA-group genes present in most tissues.

**Conclusions:**

We successfully identified SNPs (rs3128852, rs117744700, and rs28366355) associated with DKD and verified the causal association between rs3128852 and DKD. According to the in silico analysis, *TRIM27* and *HLA-A* can define DKD pathophysiology and are associated with immune response and autophagy. However, further research is necessary to understand the mechanism of immunity and autophagy in the pathophysiology of DKD and to prevent and treat DKD.

**Supplementary Information:**

The online version contains supplementary material available at 10.1186/s12916-022-02723-4.

## Background

Diabetic kidney disease (DKD) is the primary etiology of chronic kidney disease (CKD) in patients with diabetes mellitus (DM) [1] and the leading cause of CKD and end-stage renal disease (ESRD) in most developed countries [2]. Moreover, risk factors for the development of DKD in patients with type 2 DM include cardiovascular risk factors such as high urinary albumin creatinine ratio, old age, hyperglycemia, and hypertension [3]. Although the importance of hyperglycemia in the development of DKD has been illustrated in several studies [4–6], some patients with type 2 DM experience a relatively rapid deterioration in renal function, whereas others maintain normal renal function even with suboptimal glycemic levels [7]. Furthermore, patients with DKD showed familial clustering [8] and ethnic group differences [9, 10]. This susceptibility highlights the need to identify the specific genetic factors that affect the onset and progression of DKD in patients with DM.

Recently, genome-wide association studies (GWASs) have identified more than 33 genes for DKD in type 2 DM, including *APOL1*, *GABRR1*, *GCKR*, and *UMOD* [11–19]. Moreover, most of the genes reportedly associated with DKD need to be confirmed by further replication studies and a detailed analysis of their functional role in DKD using experimental models [20]. Two complex fundamental features define DKD: the decline of estimated glomerular filtration rate (eGFR) and the presence of proteinuria; hence, it would be better to combine these phenotypes during analysis, to incorporate DM and hypertension-related CKD. However, previous research on GWAS phenotypes for DKD or CKD was confined to the assessment of single phenotypes, such as uric acid, eGFR, ESRD, and proteinuria, and failed to focus on defining DKD [11–19, 21]. Hypertension is both an underlying risk factor and a consequence of DKD due to persistent high blood pressure in the arteries around the kidney [22]. Additionally, up to 75% of patients with DM also experience hypertension, and individuals with only hypertension frequently exhibit signs of insulin resistance [23, 24]. Although previous studies were singularly focused on either CKD or DKD, genes such as *UMOD* were linked to both hypertensive CKD (non-DKD) and DKD [20, 25, 26]. Hence, we hypothesized that single-nucleotide polymorphism (SNP)-related traits for DKD could be discovered through a GWAS mega-analysis using multinomial logistic regression (MLR).

In addition, although there are few studies on decreased renal function in middle Eastern descent [21], Japanese [17, 27] and Han Chinese [18] populations, most studies were conducted on the European and African-American populations [7, 12–16, 19]. There is a need for GWAS on DKD in large-scale Korean multi-cohorts. As reported in previous studies by the Veterans Health Service Medical Center (VHSMC) [28, 29], several elderly veterans are diagnosed with DKD due to the extended duration of type 2 DM. Consequently, studies on DKD in Korean multi-cohorts would be helpful. Toward this goal, we conducted a large-scale GWAS mega-analysis of multi-cohorts, combining the VHSMC cohorts and Korean Genome and Epidemiology Study (KoGES) consortium using MLR with four groups: normal, DM without CKD (“only DM”), CKD without DM (“only CKD”) and DKD.

## Methods

### Study population

**Fig. 1:**
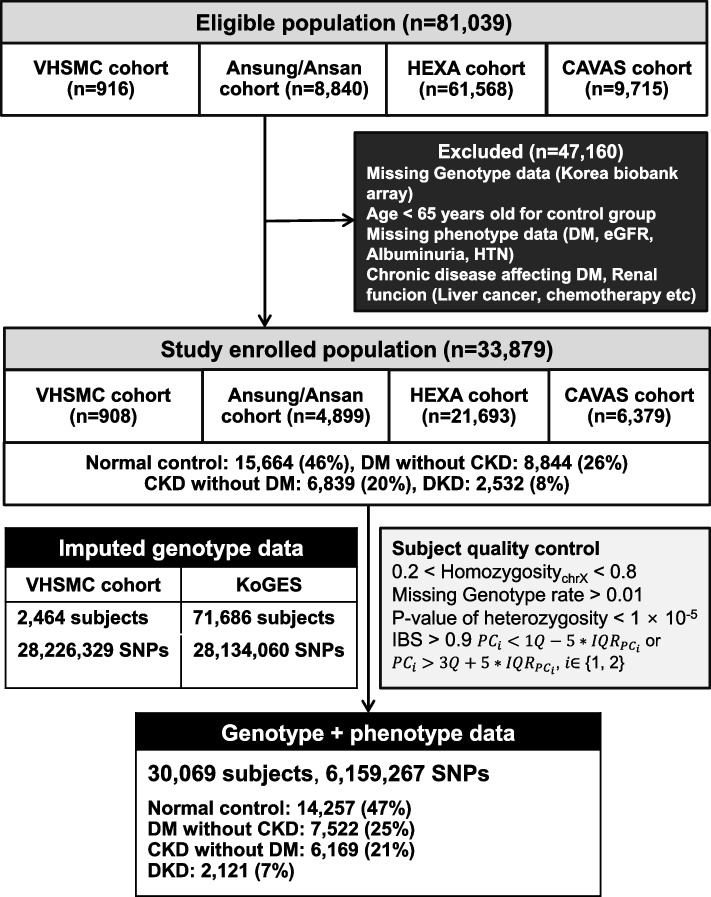
Schematic representation of the selection of the study population. VHSMC, Veterans Health Service Medical Center; KoGES, Korean Genome and Epidemiology Study; HEXA, KoGES health examinees; CAVAS, KoGES cardiovascular disease association study; SNP, single-nucleotide polymorphisms; DM, diabetes mellitus; CKD, chronic kidney disease; DKD, diabetic kidney disease; HTN, hypertension

Clinical and genetic data from multi-cohorts of the VHSMC cohort and the KoGES consortium were integrated in this study (sample size, *n* = 81,039, Fig. 1). In the previously constructed VHSMC cohort, diagnosed with type 2 DM by VHSMC endocrinologists [28, 29], those who met the inclusion criteria (*n* = 916) were enrolled in this study. The KoGES consortium is a nationwide cohort representative of genome research in Korea [30], of which three cohorts related to population-based studies [Korean Association Resource from Ansan and Ansung (KARE, *n* = 8840) cohort, KoGES Health Examinees (HEXA, *n* = 61,568) cohort, and KoGES cardiovascular disease association study (CAVAS, *n* = 9,715) cohort] were enrolled in this study. This study excluded subjects without Korea Biobank Array genotype data or phenotype (DM, eGFR, albuminuria, and hypertension) data, those who had chronic diseases affecting DM (kidney cancer, pancreatic disease, etc.) and renal function (liver cancer, chemotherapy, etc.), and those younger than 65 years for the control group from the VHSMC cohort and KoGES consortium. After applying the exclusion criteria, 30,069 participants were included in the GWAS mega-analysis (Fig. 1). The VHSMC cohort contained patients diagnosed with type 2 DM and hypertension by certified doctors, whereas in the KoGES cohort data, DM was defined by any of the following four categories (Table 1): (1) DM diagnosis checked in the questionnaire, (2) blood glucose levels ≥ 200 mg/dL 2 h after glucose loading, (3) glycosylated hemoglobin (HbA1c) amount ≥ 6.5%, and (4) overnight fasting blood glucose levels ≥ 126 mg/dL. To develop a distinct control group, participants aged 65 years or above who did not have DM or renal failure were included in the analysis.

**Table 1:** Criteria for the outcome groups for multinomial logistic regression

	Satisfied with any of the following?				
**1**	1) Checking in DM diagnosis of the questionnaire 2) 2 h loading of blood glucose levels ≥ 200 mg/dL 3) HbA1c ≥ 6.5% 4) Overnight fasting blood glucose levels ≥ 126 mg/dL	**✓**	**✓**		
**2**	1) Creatinine 1.3 mg/dL (men), 1.0 mg/dL (women) 2) eGFR < 60 mL/min/1.73 m^2^ 3) Albuminuria	**✓**		**✓**	
**Group**		DKD (1)	Only DM (2)	Only CKD (3)	Control (4)

*HbA1C* Glycosylated hemoglobin

### DKD phenotype definition

The eGFR was calculated using the abbreviated Modification of Diet in Renal Disease equation as follows: 175 × serum creatinine^−1.154^ × age^−0.203^ (×0.742 if female) [31]. According to the Kidney Disease: Improving Global Outcomes (KDIGO) guidelines, eGFR and albuminuria categories were used to assess a renal complication [32]. Albuminuria was classified into three categories based on the following albumin-to-creatinine ratios: < 3 mg/mmol creatinine, normal to mildly increased; 3–29 mg/mmol, moderately increased; and ≥ 30 mg/mmol creatinine, severely increased. Since the dysfunction of the glomerular barrier (represented by proteinuria) and reduced renal function (assessed using the eGFR) may develop independently, the various phenotypes for DKD were defined as follows (Table 1): (1) creatinine level > 1.3 mg/dL (men), > 1.0 mg/dL (women), (2) eGFR < 60 mL/min/1.73 m^2^, and (3) albuminuria.

### Ethics

The institutional review board (IRB) at the Veterans Health Service Medical Center approved the study protocols for the VHSMC cohorts after obtaining informed consent (IRB No. 2018-08-032 and IRB No. 2020-02-031). However, the IRB approved the protocol for the KoGES consortium (IRB No. 2021-05-007) after waiving the need for informed consent since this was a retrospective study; the committee of the National Biobank of Korea, the Korea Disease Control and Prevention Agency (KBN-2021-042), approved the use of bioresources in this study. This study was conducted in compliance with the Declaration of Helsinki.

### Genotyping and imputation

Genomic DNA was extracted from venous blood samples, and 100 ng DNA was genotyped using the c Affymetrix Axiom 1.1 (Affymetrix, Santa Clara, CA) [33]. The genotypes were identified using a K-medoid clustering-based algorithm to minimize the batch effect [34]. The PLINK (version 1.9, Boston, MA) and ONETOOL [35] software packages were used for quality control processes. We excluded samples matching any of the following criteria: (1) sex inconsistencies or (2) a call rate of up to 95%. Furthermore, SNPs were filtered out if (1) the call rate was lower than 95% or (2) the Hardy–Weinberg equilibrium (HWE) test showed *P* < 1×10^−5^. The genotype imputation was conducted using the Northeast Asian Reference Database imputation server (https://nard.macrogen.com/), and data of 1779 Northeast Asians [36] were used for the reference panel. Pre-phasing and imputation were performed using Eagle v2.4 [37] and Minimac4 [38], respectively. Post imputation, imputed SNPs were removed if the *R*-squared value was less than 0.8, there were duplicated SNPs, missing genotype rates were more than 0.05, *P*-values for HWE were less than 1×10^−5^, or minor allele frequencies were less than 0.01. After quality control and imputation, 6,159,267 SNPs were selected for association analyses.

### Genome-wide association analysis

Baseline characteristics of the study population have been reported as means ± standard deviation (SD) for continuous variables and numbers and as proportions for categorical variables. Genome-wide association analyses were conducted with an MLR model for the categorical response variable with four levels (normal, only DM, only CKD, and DKD), implemented in SNPTEST v2.5.6 [39]. We evaluated the overall fit of the model by comparing the likelihood of the two models: a full model with genotype risk factors and a reduced model with covariates only. Age, sex, and ten principal component scores were selected as covariates. To estimate the marginal genetic effects for six contrasts [contrast (1) only DM vs. normal, (2) only CKD vs. normal, (3) only DKD vs. normal, (4) only CKD vs. only DM, (5) DKD vs. only DM, and (6) DKD vs. only CKD], a Wald test was used. To verify that there was no confounding due to population stratification in this study, the variance inflation factor (VIF) was calculated, whereby a VIF value close to 1 indicated no genomic inflation. The regional plot for significant genetic variation was generated using the LocusZoom software with linkage disequilibrium (LD) information of East Asians from the 1000 Genomes Project [40]. The bottom panel displays gene symbols and the location within the region, derived from 1000 genomes (ASN hg19/Nov2014). The threshold for statistical significance in this model was *P* < 5.0×10^−8^, which is conventionally considered to reflect genome-wide significance. To estimate the relative proportion of phenotypic variance explained by all observed common SNPs, genome-wide complex trait analysis (GCTA v1.91.7) was used for heritability calculation [41].

### Conditional analyses

HLA has been reported to have an effect on T1D, and variants in the HLA region were adjusted to remove the confounding effects of T1D on DKD. A conditional MLR model was applied, adjusting the effect of T1D-associated HLA variants. Among 35 previously reported T1D-associated HLA variants, one SNP (rs9275490) in DR-DQ loci and one SNP (rs9271346) in non-DR-DQ loci were included as covariates for the analysis [42]. Furthermore, to determine the variants that affect DKD independently of DM and CKD, we performed additional logistic regression analyses for DKD after adjusting for the effects of DM and CKD.

### Fine-mapping analysis

To prioritize whether the variants discovered are candidate causal variants, fine-mapping analysis was performed. Among significant GWAS hits, even after adjusting for effects of T1D-associated HLA variants, DM, and CKD, only SNPs with genome-wide significantly related to DKD were used for fine-mapping analysis. For each target variant, we first selected the set of SNPs, consisting of the most significant SNP and a 100-kb window of SNPs around it. The LD matrix between SNPs was computed using PLINK (version 1.9) [43]. Using a Bayesian approach (PAINTOR method) [44], we estimated the posterior probabilities of causative SNPs at a given fine-mapping locus.

### Functional annotation analyses

The eQTL analysis was performed using the Genotype-Tissue Expression (GTEx) dataset. To identify significant eQTL genes, it was assumed that approximately 200,000 SNPs were used in the eQTL analysis considering 20,000 genes and LD block [45]. Therefore, using the Bonferroni correction, we set the significance threshold to 0.05/200,000 (=2.5×10^−7^). We used the LocusFocus tool to generate a colocalization plot, showing the lead SNP responsible for both GWAS and eQTL signals at loci [40]. GTEx version V7 and 1000 Genomes Phase 3 East Asian LD were used for this plot. The associated genes were further investigated for differently expressed genes (DEGs) in the glomeruli of patients with DM while controlling for age from the Gene Expression Omnibus (GEO) dataset (*GSE30529*). The platform used for the analysis of *GSE30529* was the GPL571 [HG-U133A_2] Affymetrix Human Genome U133A 2.0 Array, which included genes from the kidneys of ten subjects with diabetes and twelve control samples of genes from the kidneys of healthy people. Furthermore, the Search Tool for the Retrieval of Interacting Genes/Proteins (STRING) open-access database was used to identify biological functions based on the identified genes [46]. The minimum required interaction score was set as 0.4 (medium confidence). The Kyoto Encyclopedia of Genes and Genomes (KEGG) pathway enrichment analysis [47] was also conducted using the STRING database.

### Transcriptome-wide association analysis

Gene-based association analyses were performed for 7254 protein-coding genes using PrediXcan [48]. We imputed tissue-specific (whole blood) gene expression variation from GWAS summary statistics using weights derived from a reference transcriptome dataset provided by PrediXcan. Statistical analyses were performed with the “limma” package in R (http://www.bioconductor.org/) [49]. Transcriptome-wide significance was defined at *P* = 6.89×10^−6^ (0.05/7254) via the Bonferroni correction.

## Results

### Clinical characteristics of the study participants

A total of 33,879 subjects were enrolled in this study, and the baseline characteristics of the study population are presented in Table 2. Among them, 15,664 subjects (46%) were part of the control group, whereas 8844 (26%), 6839 (20%), 2532 (8%) were part of the only DM, only CKD, and DKD groups, respectively. The mean age of the multi-cohort was 66.19 years; the VHSMC cohort had the highest mean age of 73.93 years, followed by CAVAS cohort with the mean age of 68.81 years, KARE cohort with the mean age of 68.50 years, and HEXA cohort with the mean age of 64.57 years. The proportion of male subjects was 45% of the multi-cohort; most of the VHSMC cohort were male (88%), whereas the KARE, HEXA, and CAVAS cohorts had 46, 56, and 42% male subjects, respectively. The mean body mass index (BMI) for the multi-cohort was 24.47, whereas the mean BMI for each cohort showed a similar distribution—25.30, VHSMC; 24.85, KARE; 24.33, HEXA; and 24.57, CAVAS. The proportion of DM subjects in the multi-cohort was 34%, whereas in the individual cohorts, it was 86, 39, 33, and 24% in VHSMC, KARE, HEXA, and CAVAS, respectively. In addition, hypertension showed a similar trend as diabetes. The proportion of patients with hypertension in the multi-cohort was 32%, whereas in the individual cohorts, it was 83, 37, 28, and 32% in VHSMC, KARE, HEXA, and CAVAS. The mean HbA1c (%) for the multi-cohort was 6.09. The VHSMC cohort with the highest proportion of patients with DM had the highest mean HbA1c (%) at 7.63. The mean HbA1c (%) for the KARE, HEXA, and CAVAS cohorts was 6.22, 6.05, and 5.94, respectively. The mean creatinine (mg/dL) level for the multi-cohort was 0.93, with the highest mean creatinine level detected in the VHSMC cohort at 1.61. The rest of the cohorts showed a similar trend for mean creatinine (mg/dL): KARE, 1.01; HEXA, 0.86; and CAVAS, 1.00. Furthermore, the mean eGFR (mL/min/1.73 m^2^) level for the multi-cohort was 76.18. However, this biomarker varied significantly based on the individual cohorts: VHSMC, 56.12; KARE, 67.25; HEXA, 82.10; and CAVAS, 65.78. The average uric acid level (mg/dL) was 4.98 for the multi-cohort, whereas for the individual cohorts, it was 5.74, VHSMC; 5.10, KARE; 4.92, HEXA; and 5.01, CAVAS. Although the number of patients with proteinuria was 7% overall and 4% in KARE, 7% in HEXA, and 5% in CAVAS cohorts, it was significantly higher in the VHSMC cohort at 44%.

**Table 2:** Baseline characteristics of the study populations (total sample size = 33,879)

	Entire	VHSMC cohort	KoGES Consortium			***P***
			KARE cohort	HEXA cohort	CAVAS cohort	
Sample size	33,879	908	4,899	21,693	6,379	-
Age (years)	66.19 ± 8.00	73.93 ± 5.93	68.50 ± 8.40	64.57 ± 7.54	68.81 ± 7.67	<0.001^a^
Sex (male %)	15,332 (45%)	800 (88%)	2271 (46%)	12,117 (56%)	2685 (42%)	<0.001^b^
BMI (kg/m^2^)	24.47 ± 3.06	25.30 ± 2.89	24.85 ± 3.19	24.33 ± 3.02	24.57 ± 3.12	<0.001^a^
DM (%)	11,376 (34%)	777 (86%)	1916 (39%)	7158 (33%)	1525 (24%)	<0.001^b^
Hypertension (%)	10,715 (32%)	752 (83%)	1803 (37%)	6,116 (28%)	2044 (32%)	<0.001^b^
HbA1c (%)	6.09 ± 1.04	7.63 ± 1.74	6.22 ± 1.17	6.05 ± 1.03	5.94 ± 0.85	<0.001^a^
Creatinine (mg/dL)	0.93 ± 0.42	1.61 ± 1.60	1.01 ± 0.47	0.86 ± 0.39	1.00 ± 0.34	<0.001^a^
eGFR (mL/min/1.73 m^2^)	76.18 ± 16.80	56.12 ± 22.47	67.25 ± 15.12	82.10 ± 18.24	65.78 ± 12.40	<0.001^a^
Uric acid (mg/dL)	4.98 ± 1.38	5.74 ± 1.55	5.10 ± 1.45	4.92 ± 1.34	5.01 ± 1.43	<0.001^a^
Proteinuria						<0.001^b^
No	29,690 (88%)	491 (54%)	4651 (95%)	19,726 (91%)	4822 (76%)	-
Yes	2425 (7%)	403 (44%)	185 (4%)	1549 (7%)	288 (5%)	-
MLR group (%)						<0.001^b^
Normal	15,664 (46%)	72 (8%)	1968 (40%)	10,518 (49%)	3106 (49%)	-
Only DM	8844 (26%)	330 (36%)	1459 (30%)	5978 (28%)	1077 (17%)	-
Only CKD	6839 (20%)	59 (7%)	1015 (21%)	4017 (18%)	1748 (27%)	-
DKD	2532 (8%)	447 (49%)	457 (9%)	1180 (5%)	448 (7%)	-

*VHSMC* Veterans Health Service Medical Center, *KoGES* Korean Genome and Epidemiology Study, *KARE* KoGES Ansan and Ansung, *HEXA* KoGES Health Examinees, *CAVAS* KoGES cardiovascular disease association study

^a^ANOVA test, ^b^Chi-square test

### Genome-wide association analysis for DKD

**Fig. 2:**
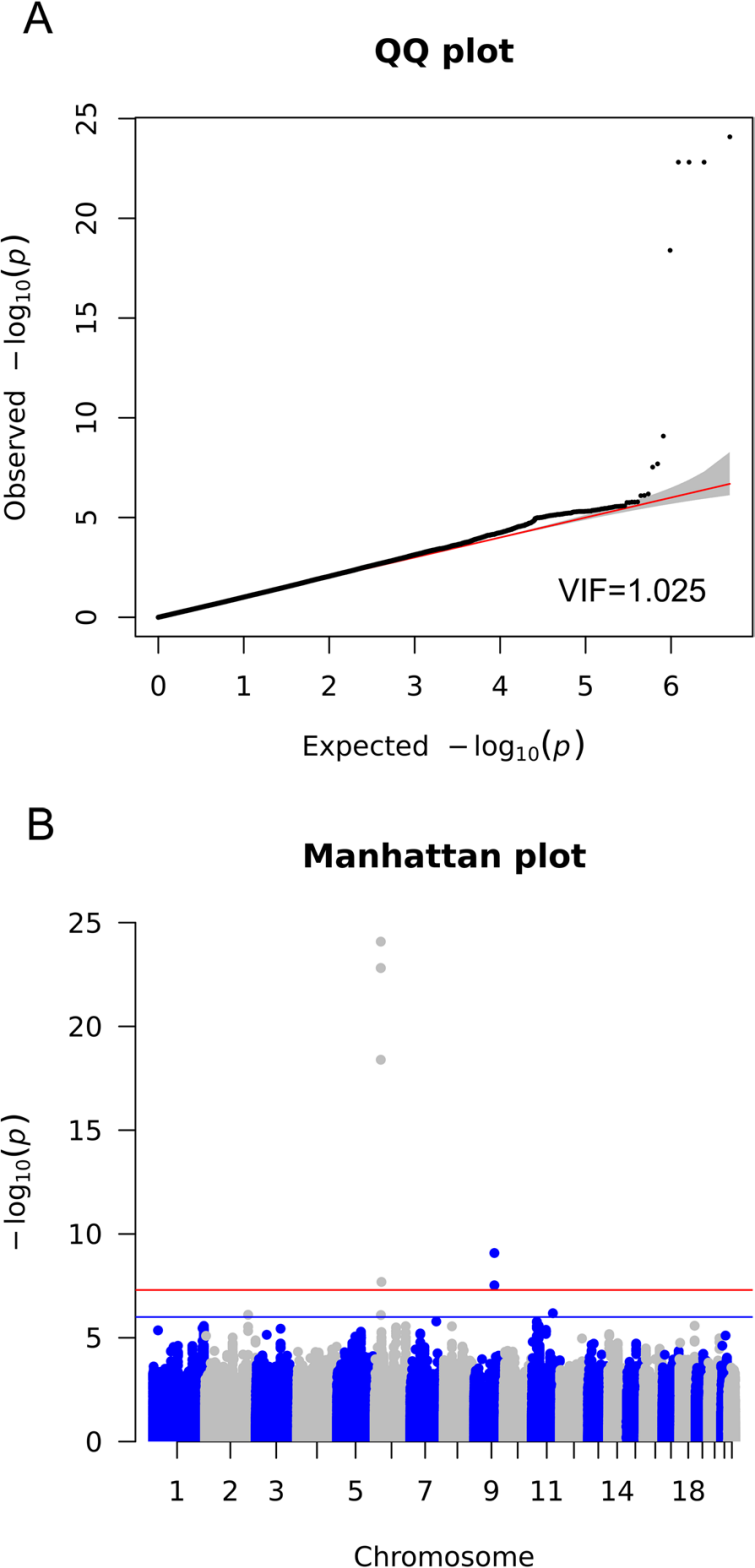
Quantile–quantile (Q-Q) and Manhattan plots for multinomial logistic regression analysis. **A** Q-Q plot showing expected vs. observed −log_10_*P*-values. The expected line is shown in red, and confidence bands are shown in gray. **B** Manhattan plot of the *P*-values in the genome-wide association study (GWAS) multinomial logistic analysis for DKD phenotype (red = genome-wide line, blue = suggestive line)

**Fig. 3:**
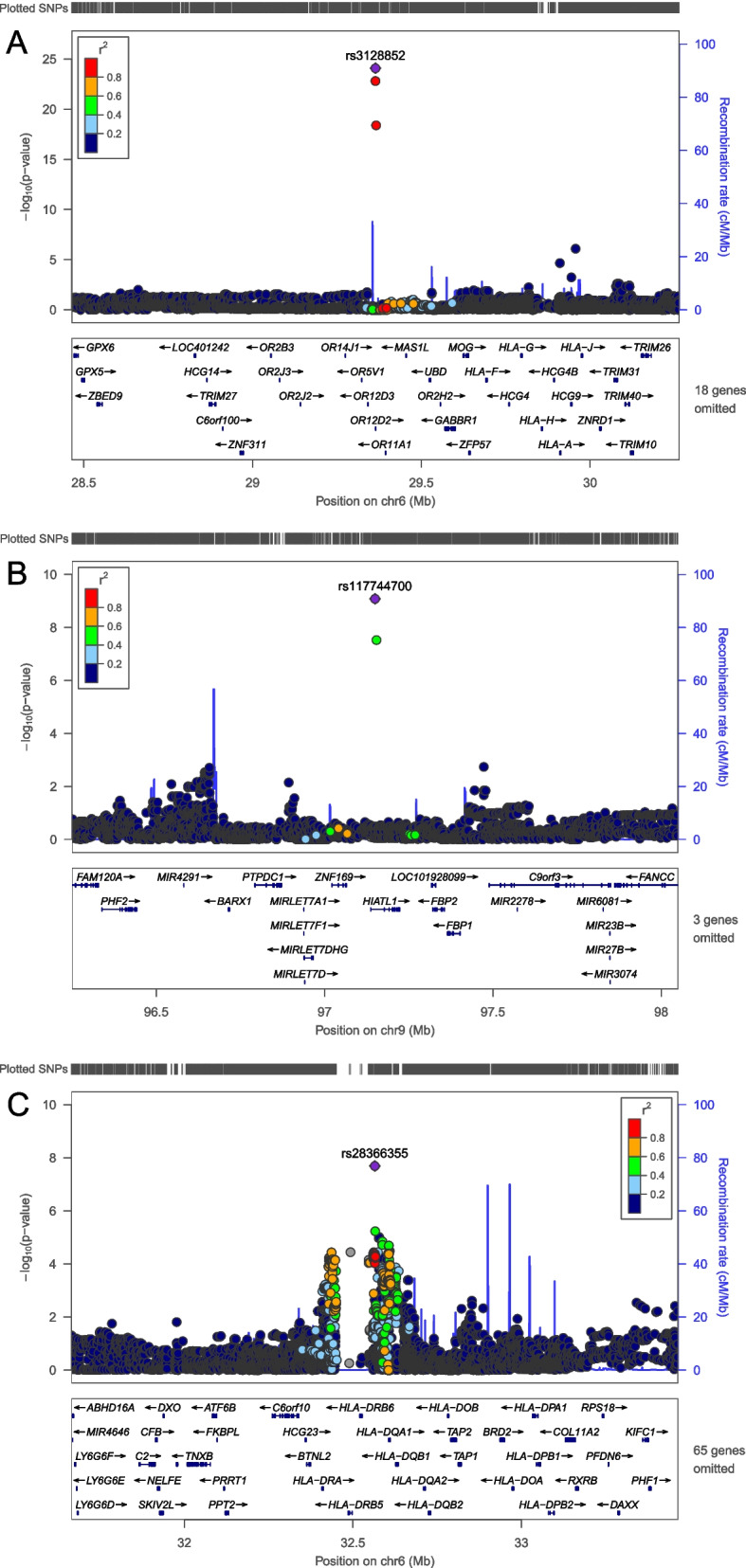
LocusZoom plots for significant single-nucleotide polymorphisms. **A** Regional plot of rs3128852. **B** Regional plot of rs117744700. **C** Regional plot of rs28366355. Vertical axis indicates the −log10 of the *P-*values, whereas the horizontal axis indicates the chromosomal position Each dot represents the single-nucleotide polymorphism (SNP) results for GWAS mega-analysis. Approximate linkage disequilibrium of East Asians from the 1000 Genome Project between the most significant SNPs are listed at the top of each plot; the other SNPs are shown by the 𝑟^2^ key in each plot

We conducted a GWAS mega-analysis using MLR on 6,159,267 SNPs from 30,069 subjects from the VHSMC cohort and the KoGES consortium, and they were analyzed after the genotype quality control (Fig. 1). First, we investigated genetic variants that significantly differed between the four groups, using the likelihood ratio test (LRT). Three genetic variants passed the genome-wide significant threshold (*P* <5.00×10^−8^) and were identified as novel variations for DKD. The most significant SNP was rs3128852 near *OR5V1* (LRT *P* = 8.21×10^−25^), followed by rs117744700 near *HIATL1* (LRT *P* = 8.28×10^−10^), and rs28366355 near human leukocyte antigens *HLA-DRB1* and *HLA-DQA1* (LRT *P* = 2.04×10^−8^; (Table 3 and Additional file 1: Table S1). Notably, rs3128852 and rs117744700 were significant in DKD even after adjusting for the association of the T1D-associated HLA variants, whereas rs28366355 in the HLA region lost significant association (Additional file 1: Table S2). Furthermore, we confirmed that rs3128852 and rs117744700 were associated with DKD independently of the link between DKD and DM or CKD (Additional file 1: Table S3). Figure 2 represents the quantile–quantile (Q-Q) plot, which verified that there was no inflation in the test statistics (VIF=1.025), and Manhattan plots of the results of the MLR GWAS mega-analysis. Regional association plots are shown in Fig. 3. In addition, two more suggestive variants (*P* <1.00×10^−6^) were identified: rs1824125 near *PGR* (LRT *P* = 6.58×10^−7^) and rs75292524 near *PARD3B* and *NRP2* (LRT *P* = 7.82×10^−7^; Table 3). The Q-Q and Manhattan plots for six contrasts for each pair are shown in Additional file 2: Fig. S1. Since the HLA region has a notably higher level of variability than the rest of the genome, it was confirmed through the multidimensional scaling (MDS) plot that the false-positive association was not caused by population stratification (Additional file 2: Fig. S2). Furthermore, fine-mapping results supported that the top SNP (rs3128852) with the highest posterior probability (almost 1.0) is likely to have a potential causal effect on DKD (Additional file 1: Table S4 and Additional file 2: Fig. S3A). However, the posterior probability of the second (rs117744700) significant variants was almost zero (Additional file 2: Fig. S3B). These SNPs may not actually be potential causal variants, or the causality may not be estimated owing to the absence of SNPs with high LD relationships around the target SNP (Fig. 3B, C). Since GWAS is designed for identifying variants associated with the phenotype of interest, and not causal variants, the top SNP can be interpreted as a potential causal SNP for DKD and a related SNP for the other two SNPs. The eQTL colocalization plots for potential causal variants of DKD are shown in Additional file 2: Fig. S4.

**Table 3:** Results of the GWAS mega-analysis using multinomial logistic regression (leading SNPs, top five)

Chr	Position	rsID	Allele^**a**^	AF	GWAS			Gene symbol
						OR (95% CI)	***P***	
6	29364135	rs3128852	C/T	0.281			**8.21*×*10**^***−*25**^	*OR5V1* (intronic)
					- only DM vs. normal	1.079 (1.030–1.132)	**5.81 × 10**^**−4**^	
					- only CKD vs. normal	0.977 (0.934–1.024)	0.342	
					- DKD vs. normal	1.421 (1.296–1.572)	**2.32 × 10**^**−24**^	
					- only CKD vs. only DM	0.905 (0.859–0.955)	2.28 ×10^−4^	
					- DKD vs. only DM	1.317 (1.226–1.416)	**4.72 × 10**^**−14**^	
					- DKD vs. only CKD	1.455 (1.351–1.567)	**3.13 ×10**^**−23**^	
9	97149910	rs117744700	G/C	0.249			**8.28*×*10**^***−*10**^	*HIATL1* (intronic)
					- only DM vs. normal	0.950 (0.911–0.993)	0.030	
					- only CKD vs. normal	0.967 (0.926–1.017)	0.216	
					- DKD vs. normal	0.769 (0.725–0.818)	**6.22 ×10**^**−11**^	
					- only CKD vs. only DM	1.020 (0.966–1.078)	0.481	
					- DKD vs. only DM	0.809 (0.745–0.879)	**5.22 ×10**^**−7**^	
					- DKD vs. only CKD	0.793 (0.729–0.863)	**7.65 ×10**^**−8**^	
6	32565056	rs28366355	G/T	0.498			**2.04*×*10**^***−*8**^	*HLA-DRB1*(dist=7443),
					- only DM vs. normal	1.117 (1.070–1.169)	**3.38** ***×*** **10**^***−*8**^	*HLA-DQA1*(dist=40127)
					- only CKD vs. normal	1.085 (1.037–1.137)	**1.46** ***×*** **10**^***−*4**^	(intergenic)
					- DKD vs. normal	1.114 (1.040–1.201)	**9.52*×*10**^***−*4**^	
					- only CKD vs. only DM	0.971 (0.925–1.018)	0.224	
					- DKD vs. only DM	0.998 (0.931–1.068)	0.941	
					- DKD vs. only CKD	1.027 (0.958–1.102)	0.448	
11	100902053	rs1824125	A/C	0.248			6.58 × 10^−7^	*PGR* (UTR3)
					- only DM vs. normal	1.075 (1.026–1.131)	1.50 × 10^−3^	
					- only CKD vs. normal	1.030 (0.981–1.084)	0.229	
					- DKD vs. normal	1.211 (1.114–1.327)	1.59 × 10^−7^	
					- only CKD vs. only DM	0.957 (0.907–1.012)	0.118	
					- DKD vs. only DM	1.126 (1.043–1.215)	2.14×10^−3^	
					- DKD vs. only CKD	1.176 (1.088–1.272)	4.58×10^−5^	
2	206490310	rs75292524	A/G	0.283			7.82×10^−7^	*PARD3B*(dist=5424),
					- only DM vs. normal	0.960 (0.921–1.002)	0.072	*NRP2*(dist=56914)
					- only CKD vs. normal	0.991 (0.947–1.039)	0.720	(intergenic)
					- DKD vs. normal	0.815 (0.768–0.867)	7.82×10^−7^	
					- only CKD vs. only DM	1.126 (1.043–1.215)	0.239	
					- DKD vs. only DM	0.848 (0.784–0.918)	4.06×10^−5^	
					- DKD vs. only CKD	0.822 (0.759–0.891)	1.59×10^−6^	

*AF* Allele frequency, *CKD* Chronic kidney disease, *Dist* Distance, *DM* Diabetes mellitus, *DKD* Diabetic kidney disease, *SNP* Single-nucleotide polymorphism, *OR* Odds ratio

^a^Major/minor allele

### Functional annotation

The most significant variant (rs3128852) was an eQTL for *TRIM27* (tripartite motif-containing 27) in whole blood (*P* = 2.10×10^−9^) and adipose-subcutaneous cells (*P* = 1.60×10^−7^; Table 4). However, the second significant variant (rs117744700) did not have any eQTL-associated genes in the GTEx database. The third significant variant (rs28366355) was an eQTL for *CYP21A1P*, *HLA-DQA1*, *HLA-DQA2*, *HLA-DQB1*, *HLA-DQB2*, *HLA-DRB1*, *HLA-DRB6*, and *XXbac-BPG154L12.4* in most tissues. The eQTL analysis results for major tissues that may be associated with renal function (whole blood; artery-aorta, artery-tibial; adipose-subcutaneous; heart-left ventricle; renal-cortex, adrenal gland) are presented in Table 4 and Additional file 1: Table S5. Colocalization plots for the top SNP (rs3128852) in the *TRIM27* and *HLA-A* gene and a third significant SNP (rs28366355) in the HLA region (from 29,677,984 to 33,485,635 bp in chromosome 6) are shown in Additional file 2: Fig. S4. Moreover, the decreased expression levels of *TRIM27* [estimate of the log2-fold-change (logFC) corresponding to the DKD control glomeruli was −0.506 and *P* = 4.10×10^−3^], *HLA-A* (logFC = −1.053 and *P* = 1.19×10^−3^), *HLA-DQA1* (logFC =−1.758 and *P* = 8.09×10^−3^), *HLA-DQB1* (logFC = −1.234 and *P* = 4.78×10^−4^), *HLA-DQB2* (logFC = −0.450 and *P* = 2.21×10^−2^), and *HLA-DRB6* (logFC =−0.725 and *P* = 3.53×10^−5^) genes were associated with DKD glomeruli in GEO datasets (*GSE30529*; Table 5 and Additional file 1: Table S6). Except for *HLA-DRB6*, which cannot be annotated in the STRING database, five genes were used as input genes in the STRING database to identify known and predicted biological functional networks. The HLA genes (*HLA-A*, *HLA-DQA1*, *HLA-DQB1*, and *HLA-DQB2*) had a strong interaction with each other (interaction score > 0.75), but *TRIM27* did not interact with any of these genes (Additional file 2: Fig. S5). Although 10 KEGG pathways were associated with this network, only two genes (*HLA-DQA1* and *HLA-A*) were annotated for DKD, which are associated with the immune response related to DKD pathogenesis (Table 6). Heritability estimates for DKD, CKD, and DM are presented in Table 7.

**Table 4:** cis-eQTLs of SNPs associated with DKD

Chr	SNP	Gene symbol	***P***	NES	Tissue
6	rs3128852	*TRIM27*	2.10×10^−9^	−0.19	Whole blood
		*HLA-A*	1.60×10^−7^	0.23	Adipose - Subcutaneous
9	rs117744700	-	-	-	-
6	rs28366355	*CYP21A1P*	1.30E−11	0.30	Whole blood
			1.50E−08	0.28	Artery - Tibial
		*HLA-DQA1*	5.40E−15	−0.34	Artery - Aorta
			5.00E−12	−0.22	Artery - Tibial
			6.20E−11	−0.27	Adipose - Subcutaneous
			6.60E−09	−0.13	Whole blood
		*HLA-DQA2*	1.00E−97	0.90	Whole blood
			6.70E−80	1.00	Adipose - Subcutaneous
			2.30E−77	0.91	Artery - Tibial
			8.50E−45	0.93	Heart - Left ventricle
		*HLA-DQB1*	4.30E−13	−0.25	Whole blood
			2.90E−11	−0.34	Adipose - Subcutaneous
			8.30E−08	−0.34	Heart - Left ventricle
			2.00E−07	−0.22	Artery - Tibial
			2.00E−07	−0.28	Artery - Aorta
		*HLA-DQB2*	2.00E−24	0.44	Whole blood
			1.90E−15	0.38	Artery - Tibial
			8.10E−15	0.46	Adipose - Subcutaneous
		*HLA-DRB1*	1.70E−25	−0.18	Whole blood
			1.90E−22	−0.24	Artery - Tibial
			5.20E−22	−0.37	Heart - Left ventricle
			1.20E−21	−0.32	Adipose - Subcutaneous
			4.70E−19	−0.31	Artery - Aorta
		*HLA-DRB6*	2.40E−95	1.00	Adipose - Subcutaneous
			4.70E−75	0.7	Whole blood
			1.20E−67	0.67	Artery - Tibial
			3.30E−41	0.71	Artery - Aorta
			3.20E−16	0.34	Whole blood
		*XXbac-BPG154L12.4*	5.30E−10	0.34	Adipose - Subcutaneous

*Chr* Chromosome, *NES* Normalized effect size

**Table 5:** Differentially expressed genes of significant loci for DKD using Gene Expression Omnibus (GEO) dataset (*GSE30529*)

Gene symbol	log FC	AveExpr	***P***-value	B
*TRIM27*	−0.506	0.066	4.10×10^−3^	−0.556
*HLA-A*	−1.053	0.071	1.19×10^−3^	−0.904
*CYP21A1P*	0.162	0.013	0.261	−5.750
*HLA-DQA1*	−1.758	0.951	8.09×10^−3^	−2.783
*HLA-DQA2*	NA	NA	NA	NA
*HLA-DQB1*	−1.234	0.307	4.78×10^−4^	−0.132
*HLA-DQB2*	−0.450	0.170	2.21×10^−2^	−3.698
*HLA-DRB1*	NA	NA	NA	NA
*HLA-DRB6*	−0.725	0.018	3.53×10^−5^	2.347
*XXbac-BPG154L12.4*	NA	NA	NA	NA

*logFC* estimate of the log2-fold-change corresponding to the effect or contrast, *AveExpr* average log2-expression for the probe over all arrays and channels, *B* log-odds that the gene is differentially expressed, *NA* not available

**Table 6:** KEGG pathway analysis results with the five genes

KEGG ID	Pathway description	Counts in network	Strength	FDR	Matching proteins in the network
hsa04612	Antigen processing and presentation	2/63	2.09	0.011	*HLA-DQA1*, *HLA-A*
hsa04940	Type I diabetes mellitus	2/39	2.30	0.011	*HLA-DQA1*, *HLA-A*
hsa05320	Autoimmune thyroid disease	2/48	2.21	0.011	*HLA-DQA1*, *HLA-A*
hsa05330	Allograft rejection	2/34	2.36	0.011	*HLA-DQA1*, *HLA-A*
hsa05332	Graft-versus-host disease	2/36	2.34	0.011	*HLA-DQA1*, *HLA-A*
hsa05416	Viral myocarditis	2/55	2.15	0.011	*HLA-DQA1*, *HLA-A*
hsa04145	Phagosome	2/142	1.74	0.024	*HLA-DQA1*, *HLA-A*
hsa04514	Cell adhesion molecules	2/137	1.76	0.024	*HLA-DQA1*, *HLA-A*
hsa05169	Epstein-Barr virus infection	2/193	1.61	0.036	*HLA-DQA1*, *HLA-A*
hsa05166	Human T cell leukemia virus 1 infection	2/211	1.57	0.039	*HLA-DQA1*, *HLA-A*

This measure describes how large the enrichment effect is. This measure describes how large the enrichment effect is. It is the ratio between the number of proteins in the network that are annotated with a term and the number of proteins that we can expect to be annotated with this term in a random network of the same size

*KEGG* Kyoto Encyclopedia of Genes and Genomes, *FDR* false discovery rate, *Strength* Log10(observed/expected)

**Table 7:** Heritability estimates for phenotypes via GCTA

	DM	CKD	DKD
V(G)/Vp	0.176	0.054	0.064
SE	0.012	0.011	0.011
LRT	36.867	1.488	6.437
*P*	6.32×10^−10^	0.111	5.58×10^−3^

*GCTA* Genome-wide complex trait analysis, *V(G)/Vp* ratio of genetic variance to phenotypic variance, *SE* standard error, *LRT* likelihood ratio test, *P P*-value, *DM* diabetes mellitus, *CKD* chronic kidney disease, *DKD* diabetic kidney disease

### Transcriptome-wide association analysis (TWAS) for DKD

mRNA expression levels for 7254 protein-coding genes were imputed for TWAS. Additional file 2: Fig. S6 presents the Q-Q plot, which verified that there was no inflation in the test statistics (VIF=1.047), and volcano plots of the results of the TWAS. None of the genes showed significant differences between the patients with DKD and the control group in the DEG analysis. Summary statistics for TWAS are provided in Additional file 1: Table S7.

## Discussion

In this study, we demonstrated that three novel SNPs (rs3128852, rs117744700, and rs28366355) are significantly linked to DKD. In particular, we noted that the potential causal relationship between rs1328852 and DKD was also confirmed through fine-mapping analysis. The functional analysis for rs3128852 suggests that *TRIM27* and *HLA-A* are potential genes for determining DKD pathophysiology. This study has elucidated the pathological mechanism of DKD through genome analysis.

Earlier, GWASs for DKD or CKD were limited to the analysis of specific phenotypes, such as uric acid, eGFR, ESRD, and proteinuria [11–19], and several key genes were identified—*UMOD*, *MANBA*, *DAB2*, and *SHROOM3* [50]. We hypothesized that comparing the genomes of patients with DKD and healthy normal individuals would reveal DM-related SNPs and CKD-related SNPs; hence, we divided our investigation into four subgroups, which were specialized for DKD phenotypes. In this study, the eQTL for rs3128852 showed substantial *TRIM27* and *HLA-A* expression, and the results of the subsequent functional genome studies supported these results.

*TRIM27* encodes the tripartite motif protein family, which is involved in a variety of biological activities that may be related to autophagy and pyroptosis [51]. Recent studies demonstrated that *TRIM27* was involved in the injury of glomerular endothelial cells in lupus nephritis (LN) through the FoxO1 pathway [52, 53] and in IgA nephropathy (IgAN) via T cell signaling [54]. Although the pathogenic processes of DKD and immune-related neuropathy such as LN and IgAN are different, the molecular pathways in cells may overlap, which supports our previous findings that suppression of the protein kinase B pathway could attenuate the damage by mediating the expression of *TRIM27* [52, 55]. Autophagy is strictly regulated to maintain an optimal balance of cellular component synthesis, degradation, utilization, and recycling of cellular components [56]. When kidney cells are exposed to stress, dysregulated autophagy may contribute to the accumulation of cellular damage, resulting in age-related kidney disease [56]. Several experimental studies have shown that autophagy is inhibited by podocytes or proximal tubule epithelial cells [57–59], which is consistent with our results. The accumulation of mitochondria plays a key role in the formation of reactive oxygen species, which activates pro-apoptotic signals and may result in hypertrophy of podocytes [60, 61], apoptosis of proximal tubular cells, and kidney fibrosis caused by the *WNT*-inducible signaling protein-1 [62]. Moreover, upregulation of nephrin expression in the glomeruli inhibits the expression of mammalian target rapamycin, which promotes progressive tubular damage [63]. These results support the notion that profound autophagy dysregulation is related to DKD [64].

Our study discovered that *HLA-A*-related genes (*HLA-A*, *HLA-DQA1*, *HLA-DQB1*, *HLA-DQB2*, and *HLA-DRB6*) were involved in the etiology of DKD. Although research has been conducted on the role of the immune system in CKD development, there are few studies on DKD; therefore, these results must be taken into account. According to previous studies [65, 66], renal function is associated with HLA type, such as HLA-A*01:01, HLA-A*03:01, and DQB1*02:01, which were related to CKD or ESRD. Furthermore, immune mechanisms may play a crucial role in DKD pathogenesis, especially leukocyte accumulation and associated molecular mechanisms [67]. Moreover, hyperglycemia-induced oxidative stress pathologically stimulates circulating immune cells, which enter the affected kidney and exacerbate tissue inflammation by producing pro-inflammatory cytokines and chemokines abundantly [68]. Furthermore, DNA methylation via the upregulated activity of DNA methyltransferase 1 revealed that inflamed memory immune cells aggravate DKD [54]. In addition, *HLA-DPA1* may be involved in immune mechanisms underlying DKD development, since it has been identified as a significant gene for DKD [69]. Nevertheless, inflammatory response is a major factor in the progression of DKD, and the immune response exacerbates inflammation, indicating that the adaptive immune response is crucial in DKD [70, 71]. Combining the relevance of immune responses in DKD and the results of this study, immune responses and autophagy may be considered as possible pathways in the pathophysiology of DKD.

The true strength of this study includes the utilization of a relatively large elderly cohort sample that provides a better DKD phenotype. This is because diabetes is an age-related disorder, and a longer duration of diabetes is connected with an increased risk of developing DKD [24]. Furthermore, our study focused on DKD using MLR and compared it with DM without CKD and CKD without DM, which have not been evaluated earlier. However, there are a few limitations of this study. First, in this study, DKD was not diagnosed through kidney biopsy, but by clinical diagnosis. In a previous genetic study of patients with DKD selected based on a definite diagnosis via a kidney biopsy [72], it was difficult to recruit a significant number of participants due to the diversity of the etiological processes of DKD, limiting the study methodology. Moreover, diagnostic kidney biopsy is rarely performed in clinical practice [73]. However, our work has overcome this limitation by using in silico analysis and generating reproducible results, minimizing this disadvantage. Second, subjects with T1D would be at greater risk of developing DKD due to longer DM duration, and the genome-wide significant SNPs can have a vertical or horizontal pleiotropic effect on T1D and DKD. For instance, rs28366355, located near HLA, was significantly associated with DKD in our analysis; this significant result can be inferred from its association with T1D. However, for rs3128852 and rs117744700 or nearby SNPs, no significant results were reported and pleiotropic effects thereof on T1D and DKD are not expected. Furthermore, the prevalence of T1D is very rare, ranging from 0.017 to 0.021% in Koreans [74]. Thus, most individuals with DKD in our analyses may not experience T1D, and there is very low chance of a confounding effect by T1D. Further studies with individuals with DKD and T1D and T2D disease status are necessary. Third, as a study design, a mega-analysis was conducted; however, the disparity in the cohort mix is limiting. Since the CAVAS cohort was established for cardiovascular disease research, it has relatively high prevalence of hypertension and non-DM-CKD. Furthermore, the VHSMC cohort is a hospital-based cohort, whereas the KoGES consortium is based on community survey results. Hence, the severity of diabetes in these cohorts is different. By contrast, the difference in diabetes severity across these cohorts might yield more relevant results when analyzed with respect to real-world data. Fourth, bioinformatics analysis revealed that certain genetic variants and metabolic pathways were related to DKD pathogenesis, but the underlying mechanism of these factors needs further investigation. Because a simple overlap of GWAS lead variants with GTEx nominal *P*-value results is expected to yield several false-positive candidate causal genes [75], we conducted a QTL colocalization analysis and TWAS. However, the results were underpowered given the sample size in our study. In the PheWAS catalog (https://phewascatalog.org/), for the SNP located in *HLA-A* (rs2860580) and its LD relationship with the top SNP (rs3128852), there is a significant association with genito-urinary phenotype (Additional file 2: Fig. S7). Further studies are required to elucidate the mechanism through which immune responses and autophagy influence DKD pathogenesis, as discovered in our study. Fifth, our study results are related to some HLA-related regions, and typically, the HLA region has a higher level of variability than the rest of the genome; thus, we need to be careful when interpreting the results. To address these concerns, we attempted to address this issue by applying an MDS plot and conducting a fine-mapping analysis. Nevertheless, the variants identified as significant genome-wide in our study were not all fine-mapped because fine-mapping analysis requires high-quality genetic data and a much larger sample size than that required for a GWAS [76].

## Conclusions

This study has demonstrated that three novel SNPs (rs3128852, rs117744700, and rs28366355) are significantly associated with DKD based on the MLR GWAS mega-analysis. Moreover, the causal relationship between rs1328852 and DKD was confirmed through fine-mapping analysis. The functional analysis of the genetic variants (rs1328852) detected has revealed that *TRIM27* and *HLA-A*, associated with immune response and autophagy, contribute to the etiology of DKD. Considering the mechanism through which immune responses and autophagy influence the pathophysiology of DKD, further research is necessary for effective prevention and treatment of DKD.

## Supplementary Information

**Additional file 1: Table S1.** Results of the GWAS mega-analysis using multinomial logistic regression (*P* < 0.00001). **Table S2.** Results from multinomial logistic regression with participant category as the outcome and adjusting for known T1D-associated HLA markers (rs9275490 and rs9271346) [42]. **Table S3.** Results of the logistic regression for the top two SNPs (rs3128852 and rs117744700) after adjusting for the effects of DM and CKD. **Table S4.** Results of fine-mapping analysis using top 3 SNPs. **Table S5.** Results of eQTL analysis using top 3 SNPs from GTEx. **Table S6.** Results of DEG analysis. **Table S7.** TWAS results for DKD.

**Additional file 2: Fig. S1.** Quantile-quantile (Q-Q) and Manhattan plots for six contrasts. **Fig. S2.** Multidimensional scaling plot for HLA region. **Fig. S3.** Posterior probability plot at a given fine-mapping locus. **Fig. S4.** eQTL Colocalization plots for potential causal SNP (rs3128852) for DKD. **Fig. S5.** STRING protein–protein interaction network of the five genes associated with DKD. **Fig. S6.** Quantile–quantile (Q-Q) and volcano plots for transcriptome-wide association analysis. **Fig. S7.** PheWAS of the SNP located in *HLA-A* (rs2860580) and its LD relationship (*r*^2^ = 0.928) with top SNP (rs3128852).

## Data Availability

The authors declare that the data supporting the findings of this study are available within the article (and its supplementary information as Additional file 1: Tables S1–S7 and Additional file 2: Figs. S1–S7). However, the raw datasets generated and analyzed during the current study are not publicly available since any data providing the genome data is considered to be personal property by the Korea Bioethics law. Access to data for research is available upon reasonable request under the permission of the National Biobank of Korea contact at (http://nih.go.kr/biobank/cmm/main/mainPage.do?/) and via e-mail (biobank@korea.kr).

## Abbreviations

CKD: Chronic kidney disease
DEGs: Differentially expressed genes
DKD: Diabetic kidney disease
DM: Diabetes mellitus
eGFR: Estimated glomerular filtration rate
eQTL: Expression quantitative trait loci
ESRD: End-stage renal disease
GEO: Gene Expression Omnibus
GTEx: Genotype-Tissue Expression
GWAS: Genome-wide association study
HbA1c: Glycosylated hemoglobin
HWE: Hardy–Weinberg equilibrium
IgAN: IgA nephropathy
KDIGO: Kidney Disease: Improving Global Outcomes
KEGG: Kyoto Encyclopedia of Genes and Genomes
KoGES: Korean Genome and Epidemiology Study
LD: Linkage disequilibrium
MDS: Multidimensional scaling
MLR: Multinomial logistic regression
SD: Standard deviation
SNP: Single-nucleotide polymorphism
STRING: Search Tool for the Retrieval of Interacting Genes/Proteins
VHSMC: Veterans Health Service Medical Center
VIF: Variance inflation factor
