# Supplementary material for: Identification of genetic variants associated with diabetic kidney disease in multiple Korean cohorts via a genome-wide association study mega-analysis
Source: BMC Med. 2023 Jan 11;21:16. doi: 10.1186/s12916-022-02723-4 (PMC9832630; doi:10.1186/s12916-022-02723-4)
Supplement: Supplementary file 2 — Additional file 2: Fig. S1. Quantile-quantile (Q-Q) and Manhattan plots for six contrasts. Fig. S2. Multidimensional scaling plot for HLA region. Fig. S3. Posterior probability plot at a given fine-mapping locus. Fig. S4. eQTL Colocalization plots for potential causal SNP (rs3128852) for DKD. Fig. S5. STRING protein–protein interaction network of the five genes associated with DKD. Fig. S6. Quantile–quantile (Q-Q) and volcano plots for transcriptome-wide association analysis. Fig. S7. PheWAS of the SNP located in HLA-A (rs2860580) and its LD relationship (r2 = 0.928) with top SNP (rs3128852). [file 12916_2022_2723_MOESM2_ESM.docx]

**Supplementary Figure S1.** Quantile-quantile (Q-Q) and Manhattan plots for six contrast

**
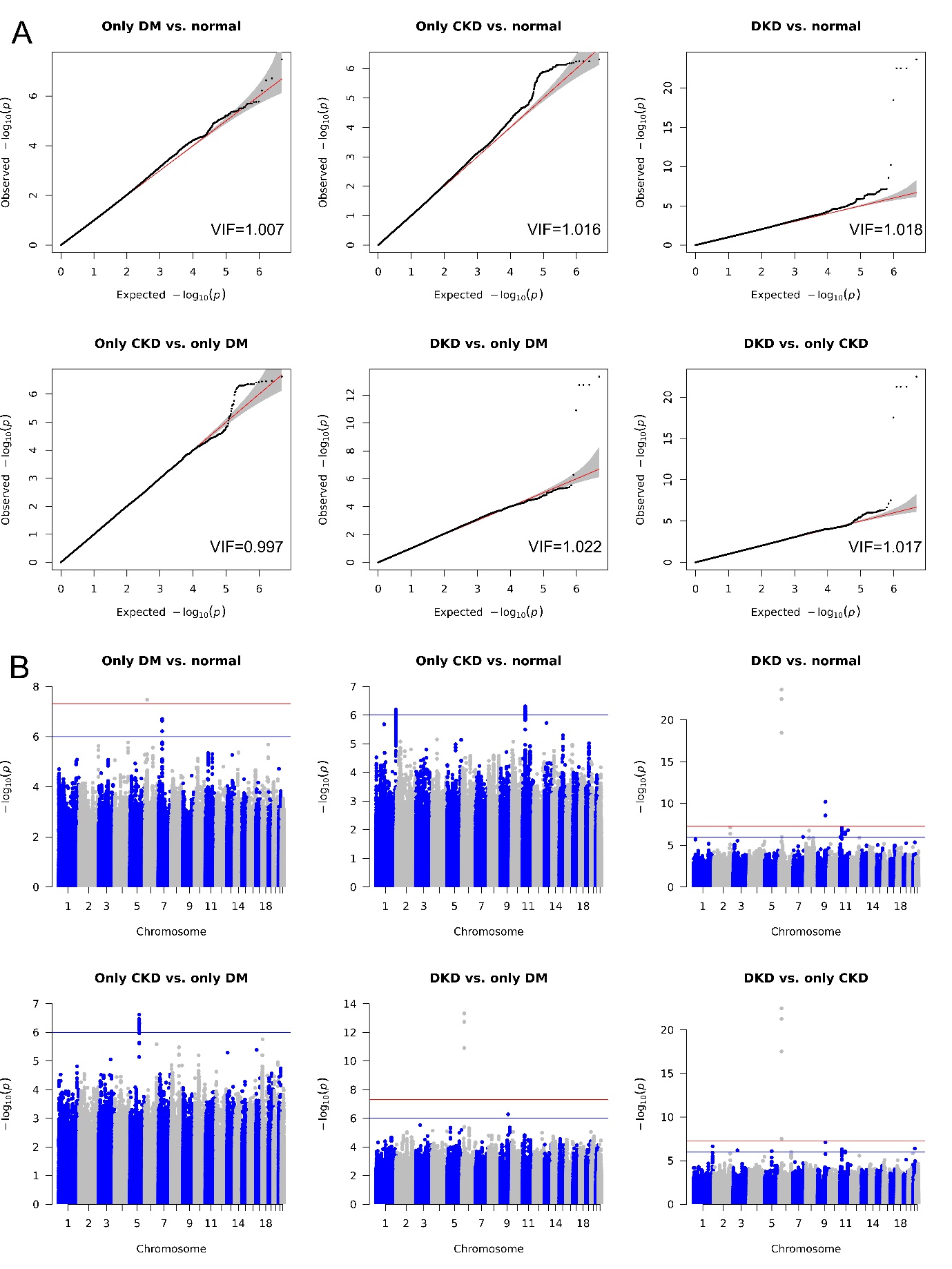
**

**A** Q-Q plot showing expected vs. observed −log_10_ *P*-values. The expected line is shown in red, and confidence bands are shown in grey. **B** Manhattan plot of the *P*-values in the genome-wide association study (GWAS) multinominal logistic analysis for DKD phenotype (red = genome-wide line, blue = suggestive line)

**Supplementary Figure S2.** Multidimensional scaling plot for HLA region

**
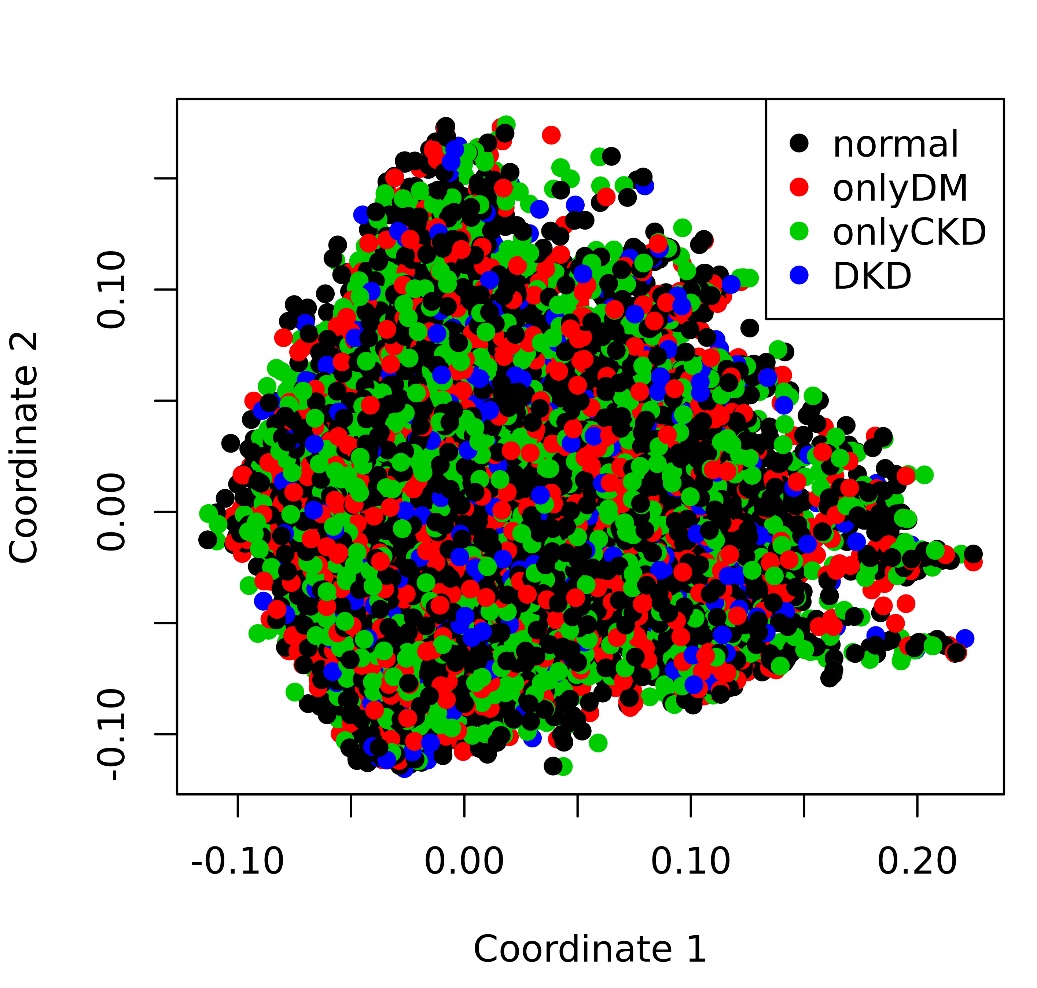
**

Multidimensional scaling (MDS) plot of human leukocyte antigen (HLA) region (from 28477 to 33448 kb). This MDS plot of 30069 samples shows that there is no population stratification.

onlyDM, diabetes mellitus (DM) without chronic kidney disease (CKD); onlyCKD, CKD without DM; DKD, diabetic kidney disease.

**Supplementary Figure S3.** Posterior probability plot at a given fine-mapping locus


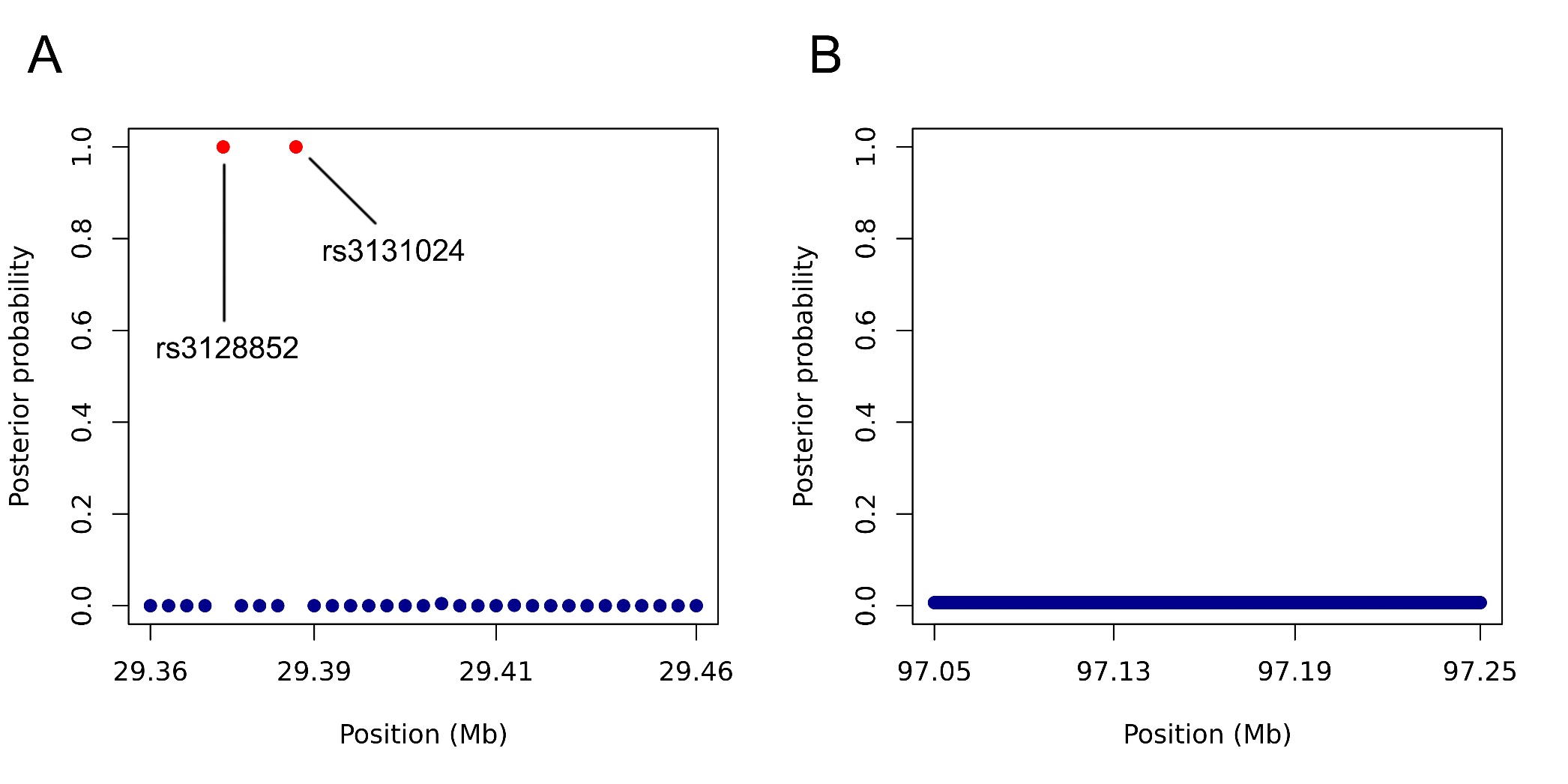


Posterior probabilities plot for the top three GWAS hits. **A** 100-kilobase window of SNPs around rs3128852 in chromosome 6 **B** 100-kilobase window of SNPs around rs117744700 in chromosome 9.

**Supplementary Figure S4.** eQTL Colocalization plots for potential causal SNP (rs3128852) for DKD

**
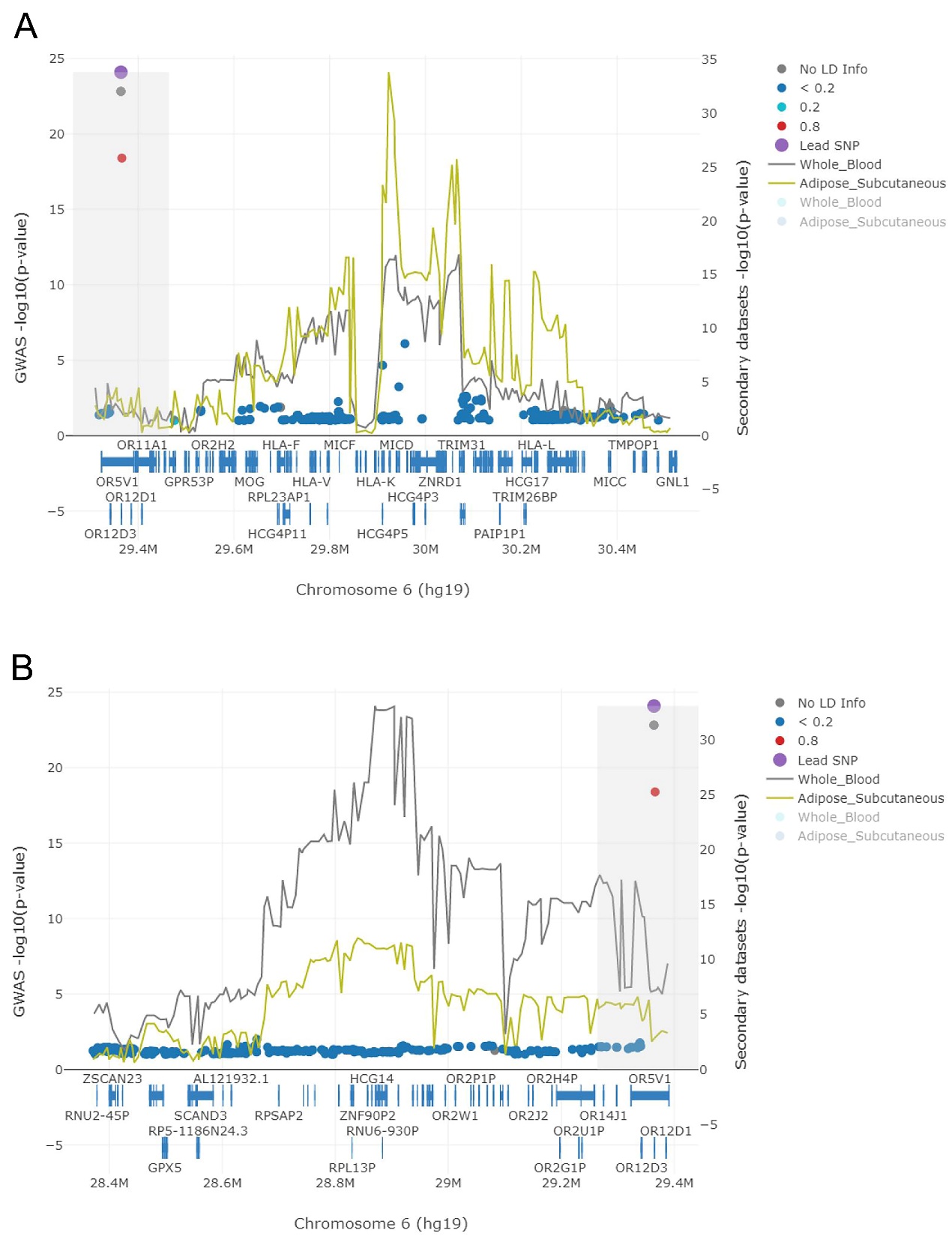
**

GWAS summary statistics of the DKD mega-GWAS analysis and whole blood and subcutaneous adipose samples from GTEx were selected for the colocalization analysis at the **A** *HLA-A* locus (bp: 29,909,037–29,913,661) and **B** *TRIM27* locus (bp: 28,903,002–28,923,988). The eQTL results of the corresponding genes are drawn as lines, and the genes shown under the x-axis represent the genes around them. The traces in the legend indicate the eQTL scatterplots that are hidden in this plot.

**Supplementary Figure S5.** STRING protein–protein interaction network of the five genes associated with DKD


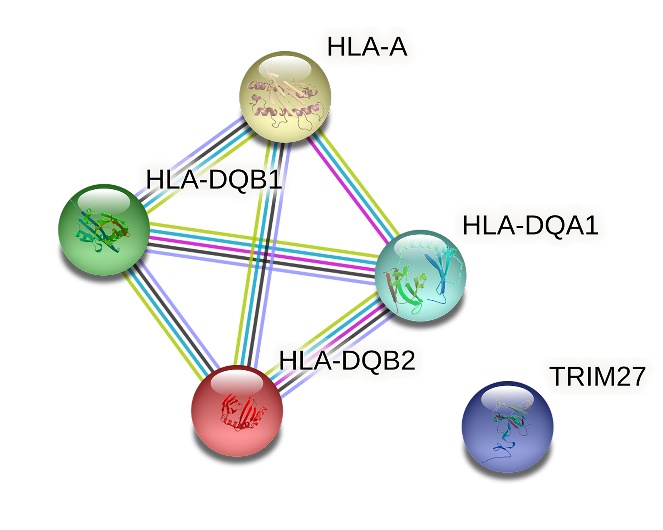


Network nodes (colored circles) represent proteins; a single node represents all the proteins produced by a single protein-coding gene, including splicing isoforms alternative polyadenylation forms. Colored lines between the nodes (edges) indicate the neighborhood of genes (green line), experimental evidence (purple line), databases (light blue line), protein homology (light purple line), and co-expression in the same or other species (black line).

**Supplementary Figure S6.** Quantile–quantile (Q-Q) and volcano plots for transcriptome-wide association analysis


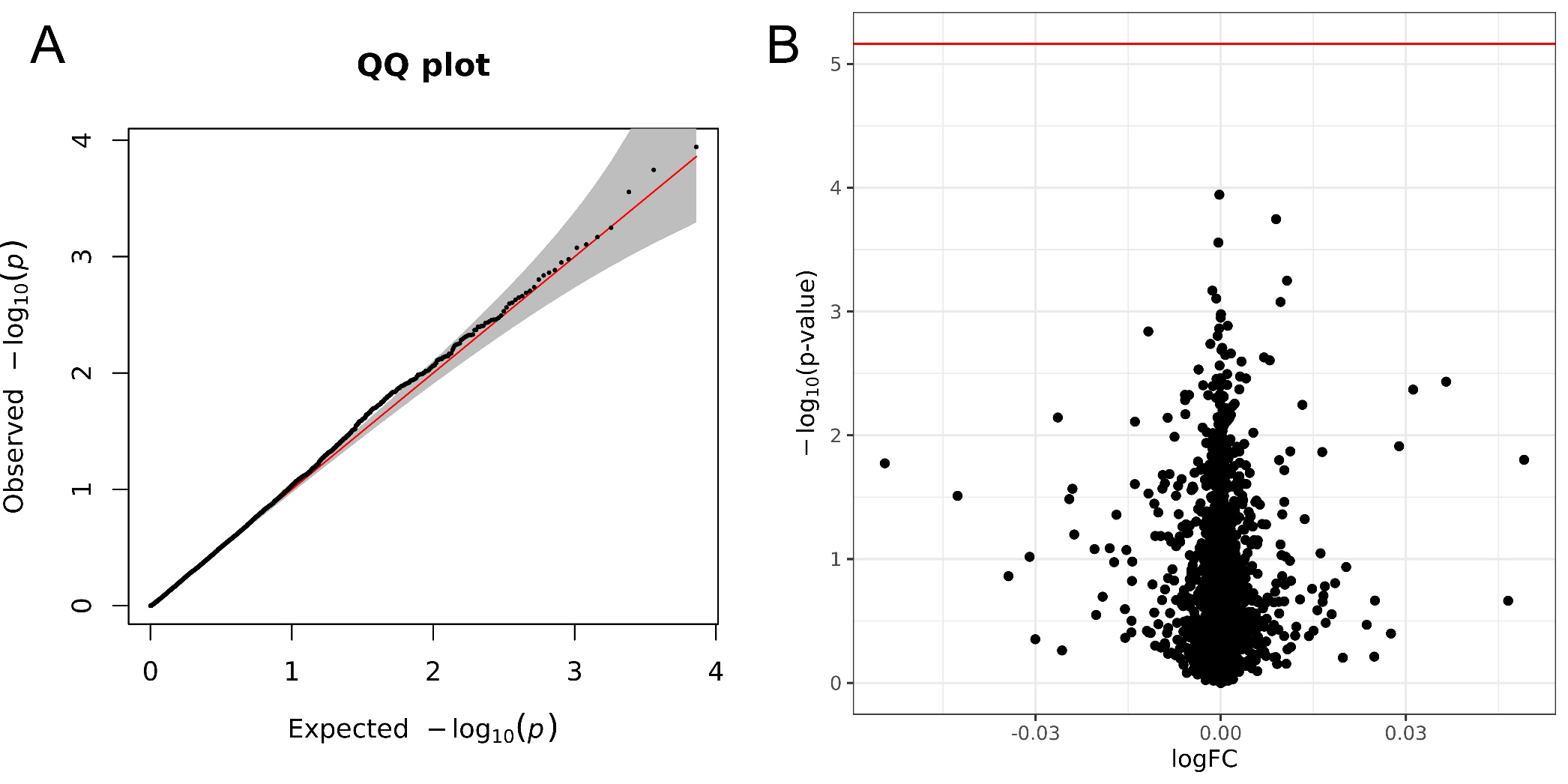


**A** Q-Q plot showing expected vs. observed −log_10_ *P*-values. The expected line is shown in red. **B** Volcano plot of the *P*-values in the transcriptome-wide association study (TWAS) for DKD phenotype (red = transcriptome-wide line).　The x-axis shows log2fold-changes in expression and the y-axis the log odds of a gene being differentially expressed.

**Supplementary Figure S7.** PheWAS of the SNP located in *HLA-A* (rs2860580) and its LD relationship (r^2^=0.928) with top SNP (rs3128852).


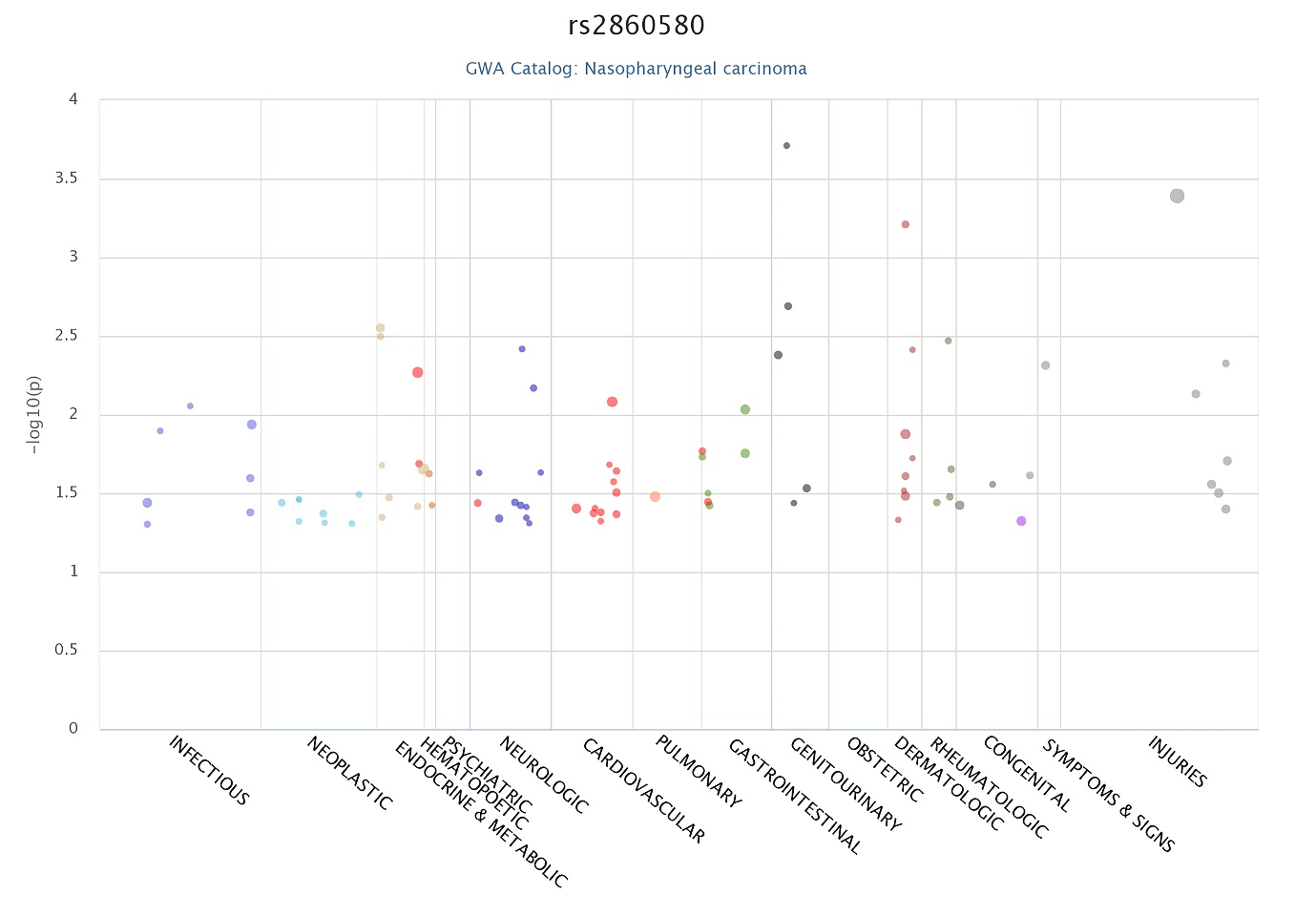


There is a significant association with genito-urinary phenotype
